# A Contemporary Review of Resectability and Treatment Strategies for Colorectal Liver Metastases: Part 1—Diagnosis, Defining Resectability, and Management of Upfront Resectable and Synchronous Disease

**DOI:** 10.3390/cancers18193165

**Published:** 2026-10-01

**Authors:** Jennifer A. Kalil, René Adam, Nicholas Meti, Peter Metrakos

**Affiliations:** 1 Cancer Research Program, Research Institute of the McGill University Health Center (RI-MUHC), Montreal, QC H4A 3J1, Canada; peter.metrakos@mcgill.ca; 2 Department of Surgery, McGill University Health Center, Montreal, QC H4A 0B1, Canada; 3 Henri-Bismuth Hepatobiliary Center, Paul-Brousse Hospital, Assistance Publique-Hôpitaux de Paris, Paris-Saclay University, 94804 Villejuif, France; rene.adam@aphp.fr; 4 Gerald Bronfman Department of Oncology, McGill University Health Center, Montreal, QC H4A 0B1, Canada; nicholas.meti@mcgill.ca

**Keywords:** colorectal liver metastases, liver resection, synchronous metastases, perioperative chemotherapy, tumor biology, surgical margins

## Abstract

**Background/Objectives**: Colorectal liver metastases (CRLMs) remain a major determinant of long-term outcome. Although hepatic resection offers the best chance of durable survival, the definition of resectability has evolved beyond technical feasibility alone. This review aims to provide a structured overview of contemporary management strategies for patients with upfront technically resectable or synchronous CRLM, with emphasis on patient selection, tumor biology, perioperative systemic therapy, margin strategy, and surgical sequencing. **Methods**: A structured literature search of PubMed/MEDLINE, Embase, Scopus, and the Cochrane Library was performed to identify studies published between 2000 and 2026. Eligible studies evaluated surgical, liver-directed, or systemic treatment strategies for CRLMs and reported outcomes including overall survival, disease-free survival, recurrence, margin status, or perioperative morbidity. Given the heterogeneity of the available evidence, findings were synthesized narratively. **Results**: Contemporary management of CRLMs requires integration of patient fitness, technical resectability, and tumor biology. In upfront technically resectable disease, routine neoadjuvant chemotherapy has not consistently improved overall survival, whereas selected high-risk patients may benefit from short-course preoperative therapy. Margin strategy is increasingly biology-informed rather than based on a universal margin width. In synchronous disease, no single operative sequence is superior for all patients; treatment should be selected according to disease burden, operative complexity, and the need to preserve access to systemic therapy. **Conclusions**: Management of upfront resectable and synchronous CRLMs should be individualized through multidisciplinary assessment. Current evidence supports a risk-adapted strategy that integrates technical feasibility, patient factors, and tumor biology while recognizing persistent uncertainty regarding optimal chemotherapy timing and surgical sequencing.

## Simple Summary

Colorectal cancer commonly spreads to the liver, and surgery remains the treatment most strongly associated with long-term survival when all visible disease can be removed. However, deciding which patients should undergo surgery has become increasingly complex. Treatment decisions now depend not only on whether the liver tumors can technically be removed, but also on the patient’s overall fitness, tumor biology, response to treatment, and whether disease is present outside the liver. This review summarizes the current approach to patients with colorectal liver metastases who present with technically resectable disease or synchronous metastases. It highlights how modern care requires individualized decision-making by multidisciplinary teams and emphasizes areas where evidence remains uncertain, particularly regarding chemotherapy timing, surgical margins, and sequencing of colorectal and liver surgery.

## 1. Introduction

Colorectal cancer is one of the leading causes of cancer-related mortality worldwide [1]. The liver is the most common site for metastatic spread, with approximately 50% of patients developing colorectal liver metastases (CRLMs) [2,3]. Hepatic resection remains the only treatment modality offering long-term survival in patients with CRLMs, with overall 5-year survival rates of 40–50% [4]. Unfortunately, recurrence rates approach 70%, with approximately 75% occurring in the first two years after surgery and the liver representing the most common site of recurrence [4,5].

The evolution of systemic therapy regimens has not only contributed to improved disease-free survival (DFS) when given perioperatively but has functioned as conversion therapy, enabling downstaging and resection in patients initially deemed unresectable [6,7,8]. In parallel, treatment modalities have expanded to include local ablative therapies, intra-arterial treatments, radiation-based approaches, and strategies to augment the future liver remnant [9,10,11,12]. Collectively, this expanded armamentarium broadens candidacy and reshapes how care is sequenced, highlighting the need for a structured framework and exposing where the evidence remains limited.

Existing consensus statements and society guidelines provide structure to the complex management of CRLM, yet many recommendations remain necessarily permissive, reflecting heterogeneity in patient presentation, institutional capability, and the predominance of nonrandomized evidence [13,14,15,16,17,18,19].

This two-part comprehensive narrative review aims to provide a structured, evidence-based overview of the current management of CRLMs and organize treatment strategies by the determinants of resectability, focusing on patient factors, tumor biology, and technical feasibility. Part 1 focuses on initial imaging and staging, the framework for defining resectability, the management of upfront technically resectable disease, and the approach to synchronous CRLM, including perioperative systemic therapy, margin strategy, and surgical sequencing. Part 2 addresses the management of borderline resectable, initially unresectable, and never resectable disease, including strategies for future liver remnant augmentation, conversion chemotherapy, liver-directed therapies, liver transplantation, and emerging approaches to disease control [20].

## 2. Methods

This work was conducted and is reported as a narrative review. A structured literature search of PubMed/MEDLINE, Embase (Ovid), Scopus, and the Cochrane Library was performed to identify relevant studies published in English between 2000 and 2026, to ensure comprehensive coverage and evidence was integrated through narrative synthesis. The search combined subject headings (MeSH) and free-text keywords related to colorectal cancer liver metastases and their management. MeSH terms included but were not limited to colorectal neoplasms, liver neoplasms/secondary, hepatectomy, ablation techniques, embolization, chemotherapy, recurrence, and treatment outcome. Additional keywords included colorectal, colon, rectal, CRLM, liver/hepatic metastas*, hepatic arterial infusion (HAI), conversion therapy, survival, DFS, recurrence, morbidity and mortality.

Sources were considered relevant if they addressed the management of CRLMs, including surgical resection, liver-directed therapy, and perioperative or conversion systemic therapy, and reported at least one of the following endpoints: overall survival (OS), DFS, recurrence patterns, margin status, and periprocedural morbidity/mortality.

The structured search was supplemented by review of reference lists from included articles, relevant clinical practice guidelines, and consensus statements, to ensure comprehensive coverage of the topic. A limited number of seminal works published before 2000 were additionally included where they remain foundational to current prognostic, patient-selection, or surgical concepts.

Given the breadth of the topic and the heterogeneity of the available evidence, which spans randomized controlled trials, prospective cohort studies, retrospective analyses, meta-analyses, and expert consensus, a narrative synthesis was used to integrate findings across surgical, liver-directed, and systemic treatment strategies. The methodological quality of this review was assessed using the Scale for the Assessment of Narrative Review Articles (SANRA) [21]. The completed SANRA worksheet is provided in Supplementary Table S1.

## 3. Initial Imaging and Staging

One of the first steps in the management of patients with suspected or known CRLMs is a detailed assessment of the hepatic tumor burden and the extent of extrahepatic disease. Cross-sectional imaging defines lesion number, size, and distribution while delineating relationships to critical structures and guiding surgical planning. In practice, contrast-enhanced computed tomography (CT) of the chest, abdomen, and pelvis serves as a readily available, first-line, whole-body staging study [22]. On the other hand, magnetic resonance imaging (MRI) is enhanced by contrast agents and multiphasic imaging protocols that improve its sensitivity and ability to detect small lesions, including those less than 10 mm [23,24]. Hepato-biliary contrast agents, such as gadoxetic acid (Gd-EOB-DTPA), provide superior lesion detection during the hepatobiliary phase by highlighting liver-specific uptake and excretion patterns [24]. Additionally, diffusion-weighted imaging (DWI) and dynamic contrast-enhanced sequences further enhance MRI’s ability to distinguish benign from malignant lesions [23,24].

In a meta-analysis comparing the diagnostic performance of CT, MRI, and fluorine 18 fluorodeoxyglucose (FDG) positron emission tomography (PET), CT achieved a 74.4% per-lesion and 83.6% per-patient sensitivity; however, its sensitivity fell to 47.7% for lesions < 1 cm [23]. By contrast, MRI shows higher sensitivity on both analyses—80.3% per lesion and 88.2% per patient—and detects subcentimeter lesions with 60.2% sensitivity [23]. DWI sequences accentuate tissues in which water movement is restricted, a feature of densely cellular metastases, thereby improving lesion detectability against a normal liver [22]. When incorporated into the MRI protocol, DWI improves lesion-level sensitivity to approximately 92% [22,24]. However, CT is often preferred for operative planning as it offers rapid acquisition, reduced susceptibility to motion artifacts, and the ability to produce high-quality multiplanar reformatted and three-dimensional images, which are essential for detailed vascular evaluation [24]. In the ideal setting, combining both modalities provides the most accurate assessment of the number, size, and distribution of liver metastases and offers a detailed anatomic roadmap that supports precise and effective preoperative planning.

FDG-PET is the most sensitive imaging modality for detecting CRLMs on a per-patient basis; however, its limited spatial resolution, inferior anatomic detail, and reduced sensitivity for subcentimeter lesions make it unsuitable as a first-line imaging study [23,24]. Although frequently performed in combination with CT (FDG-PET/CT) to improve anatomic localization, this approach does not fully overcome the intrinsic limitations of PET, and physiologic hepatic uptake can still obscure small lesions [24]. In contrast, FDG-PET/CT contributes to the detection of occult extrahepatic disease, which may not be visible on conventional imaging, but its routine use has limited impact on surgical management in patients otherwise deemed resectable [24,25]. A randomized clinical trial evaluating the utility of PET-CT prior to liver resection for CRLMs demonstrated the addition of PET/CT changed the management in only 8% of cases and did not improve DFS or OS [25].

## 4. Defining Resectability

**Figure 1:**
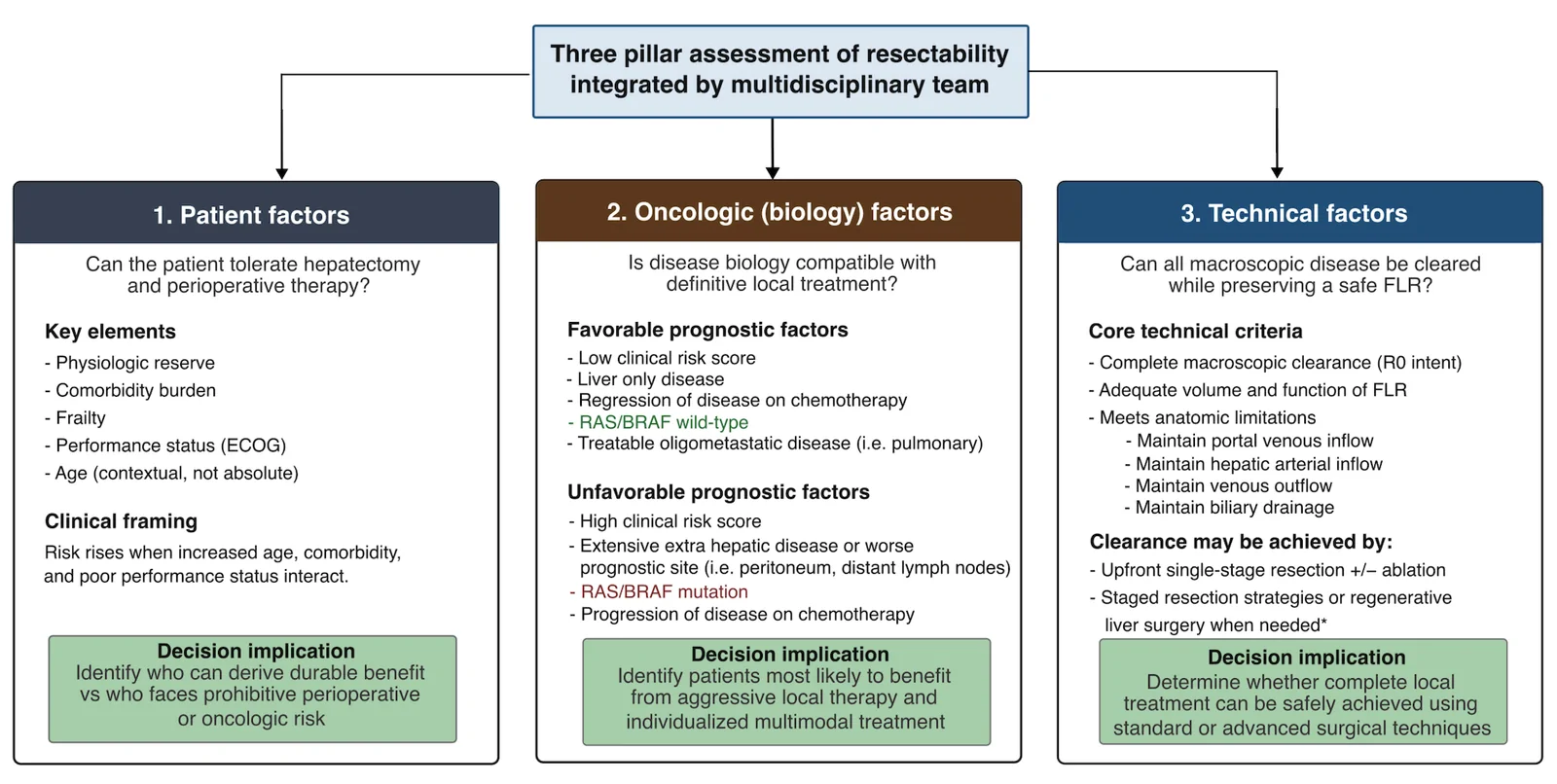
Framework for assessing resectability in CRLM. Incorporating patient, oncologic, and technical factors with multidisciplinary team evaluation is essential in determining resectability. ECOG, Eastern Cooperative Oncology Group performance status scale; FLR, future liver remnant. * Staged resection strategies as well as regenerative and FLR-augmentation procedures are discussed in Part 2 of this review.

When determining resectability, there are typically three criteria that must be considered (Figure 1). First, patient factors, such as physiologic reserve, comorbidity burden, frailty, and performance status, are critical for evaluating whether the patient can tolerate the risks of hepatectomy and perioperative therapy. Second, technical resectability must be assessed to ensure macroscopic disease clearance is technically feasible while leaving a sufficient future liver remnant (FLR). Third, tumor biology must be thoroughly understood to guide management strategies. Each of these factors plays a key role in defining overall resectability. Decisions are best made by a multidisciplinary team with specialists meeting regularly in a tumor board setting to review cases and agree on treatment intent, sequencing, approach and timing [15].

### 4.1. Patient Factors

Patient selection is critical in ensuring the best perioperative and oncologic outcomes. Advanced age, comorbidities, frailty, and functional performance each increase perioperative risk and modify outcomes after hepatectomy, yet none constitutes an absolute contraindication in appropriately selected patients. In a population-based cohort, patients > 65 years experienced higher 90-day postoperative mortality after hepatectomy for CRLMs and inferior 5-year OS compared to younger patients [26]. This group also had a greater comorbidity burden, which correlated with worse survival [26]. By contrast, when baseline differences are balanced, age alone appears to be less prognostic. In a propensity score-matched analysis limited to liver-only disease and adjusted for comorbidities, tumor burden, and perioperative treatment, patients ≥ 70 years had perioperative and long-term outcomes comparable to those < 70 years [27].

Other institutional series suggest the differences in outcomes in older individuals may reflect variability in multidisciplinary management. Patients older than 75 are less likely to receive adjuvant chemotherapy, often due to poorer performance status, and are less frequently offered repeat hepatectomy for intrahepatic recurrence—both factors contributing to poorer disease-specific outcomes [26,28]. Taken together, these data indicate that age, comorbidity, and performance status interact to shape operative risk and benefit. Resectability decisions should therefore integrate these factors to identify patients most likely to achieve durable outcomes and to recognize those at elevated perioperative and oncologic risk.

### 4.2. Technical Factors

Technical resectability refers to the ability to achieve complete macroscopic clearance of disease with an adequate FLR while preserving two contiguous hepatic segments, maintaining portal venous and hepatic arterial inflow, venous outflow, and biliary drainage in the absence of uncontrolled extrahepatic disease [29]. The adequate volume of FLR is stratified by parenchymal quality: approximately 20–25% of total liver volume in normal livers, 30% after extensive preoperative chemotherapy, and at least 40% in cirrhosis or significant fibrosis [30,31]. Additionally, the quality of the non-tumoral parenchyma is itself a determinant of safe resection, as underlying fibrosis, cirrhosis, or steatohepatitis impair regeneration and increase risk of post-hepatectomy liver dysfunction. When volumetry alone is insufficient, particularly after chemotherapy-associated injury, functional assessment with Tc-mebrofenin hepatobiliary scintigraphy or an FLR-to-body-weight ratio can further refine remnant adequacy [32]. This can typically be achieved with a single stage operation or ablation in patients with upfront resectable presentations; however, less than 20% of patients present this way [33]. By contrast, most patients present with borderline or potentially resectable disease, where technical control often depends on a combination of staged strategies or measures to augment the FLR with or without downstaging chemotherapy [29,33,34].

With technical feasibility confirmed, candidacy is further refined with assessment of tumor biology, which helps guide decisions regarding perioperative therapy, margin strategy, and treatment sequencing.

### 4.3. Tumor Biology Factors

Oncologic resectability incorporates features of increased biological risk and aids in treatment sequencing decisions rather than relying solely on technical resectability [14,17,33]. Several validated clinical risk scores (CRSs) stratify patients based on their likelihood of recurrence and, in turn, can inform treatment planning. Although their components differ slightly, the scores emphasize similar adverse features, including multiple hepatic metastases, largest lesion ≥ 5 cm, synchronous metastases or those diagnosed within 2 years of primary diagnosis, carcinoembryonic antigen (CEA) level > 200 ng/mL at diagnosis, and high-risk primary tumor characteristics including advanced stage, poor differentiation and N2 nodal status (Table 1) [35,36,37,38]. Beyond scored clinical variables, primary and metastatic tumor grade carries independent prognostic weight. Poor differentiation, including poorly differentiated clusters and high tumor budding, is consistently associated with worse survival after CRLM resection.

Notably, molecular markers—particularly RAS and BRAF mutation status—and response to chemotherapy are not included in these scoring systems but carry independent prognostic value and should be interpreted in clinical risk stratification. Patients harboring a combination of these adverse factors are generally considered to have higher clinical risk. In this setting, more recent evidence favors risk-adapted management incorporating neoadjuvant therapy rather than a standardized perioperative approach. Taken together, unfavorable biology encompasses adverse CRS features, poorly differentiated or high-grade histology, and adverse molecular alterations, whereas favorable biology reflects the absence of these features together with responsiveness to chemotherapy [39]. Tumor genotype is not the only biological determinant of outcome. The density and organization of tumor-infiltrating lymphocytes carry independent prognostic value after CRLM resection but remain investigational and are not yet incorporated into resectability algorithms [40].

#### 4.3.1. Molecular Profiling and Co-Mutations

Beyond single-gene assessment, co-occurring mutations carry additive prognostic significance. The RAS/TP53 co-mutation, present in approximately 30% of resected CRLMs, is independently associated with worse OS (HR 2.62, *p* = 0.002), a persistent deleterious effect on conditional DFS that does not attenuate over time, and a nearly three-fold increased risk of micrometastases beyond the visible tumor margin [41,42]. Incorporating SMAD4 status alongside RAS and TP53 further improves stratification beyond RAS status alone [43,44]. An international validation study across 11 tertiary centers confirmed that certain combinations of high-risk KRAS or BRAF V600E mutations with adverse clinicopathological features identified patients with virtually no long-term survival after hepatectomy, raising the question of whether surgery is biologically futile in these subgroups [45]. Whole-exome and whole-genome sequencing and large next-generation sequencing panels can refine prognostication beyond canonical drivers through pathway-centric classification, but these approaches are not yet routinely used to determine resectability or to modify surgical margins [46].

#### 4.3.2. Extrahepatic Disease

From an oncological perspective, the extent and location of extrahepatic disease are key determinants of curative potential when all disease sites are amenable to definitive local treatment [15]. The classification of oligometastatic disease, which is characterized by limited metastases in two to three sites, is associated with the possibility of local treatment and curative potential. This is in contrast with widespread extrahepatic involvement, which is generally associated with poor prognosis and unlikely to be compatible with curative strategies [15]. Importantly, the prognostic implications differ substantially by metastatic site, warranting a site-specific approach to clinical decision making.

##### Pulmonary Metastases

The lungs are the most common site of extrahepatic metastases [47]. In select patients who undergo sequential or staged resection of both hepatic and pulmonary metastases, a 5-year OS of approximately 40 to 60 percent has been reported across several studies, indicating durable outcomes are attainable when disease clearance is feasible [48,49,50,51]. In the LiverMetSurvey registry, patients who underwent resection of both liver and lung metastases achieved a 5-year OS of 44.5%, comparable to 51.5% for isolated liver metastases, whereas patients with resected liver metastases and unresected lung metastases had a 5-year OS of only 14.3% (*p* = 0.001) [52]. Additional evidence demonstrates that patients treated with liver and lung resection have a significant survival advantage compared to patients receiving systemic therapy alone (median OS: 5.58 years vs. 2.35 years, *p* < 0.0001), further supporting the local treatment of oligometastatic disease [53].

##### Peritoneal Metastases

Peritoneal metastases represent a biologically distinct and prognostically unfavorable pattern of spread. In a pooled analysis of individual patient data from 14 phase III trials (ARCAD database, n = 10,553), patients with isolated peritoneal metastases had significantly worse OS than those with isolated liver or lung metastases, which served as the reference group (adjusted HR 1.33, 95% CI 1.10–1.60; *p* = 0.003) [54]. For patients with isolated peritoneal disease amenable to complete cytoreduction, CRS can achieve a median OS of 35–40 months, though the PRODIGE-7 trial demonstrated no additional benefit from hyperthermic intraperitoneal chemotherapy (HIPEC) [55].

##### Portal, Retroperitoneal, and Para-Aortic Lymph Node Metastases

Lymph node metastases are well-documented to be associated with poor outcomes after liver resection for CRLM; however, the specific site of lymph node involvement further influences survival outcomes [47,56,57]. Historically, lymph node involvement limited to the hepatoduodenal ligament has been associated with more favorable outcomes, with reported 5-year OS rates of 18–30%, whereas involvement of the celiac axis or para-aortic regions was associated with virtually no long-term survivors [57,58]. However, with modern chemotherapy, more recent data has challenged the notion that all non-portal lymph node involvement precludes benefit from surgery. Several retrospective series have demonstrated that while long-term survival is significantly worse than in patients without lymph node metastases, a 5-year OS of 18–20% may be achieved in select patients with celiac or para-aortic lymph node involvement, although these results likely reflect the selection of patients with otherwise favorable disease profiles [59,60,61].

While extrahepatic disease complicates the management of CRLM, it does not necessarily preclude curative intent. Combining systemic therapy, local ablative techniques, and surgery can allow selected patients with limited extrahepatic involvement to achieve meaningful long-term survival when management is coordinated within a multidisciplinary team and all disease sites are amenable to definitive treatment.

## 5. Management of Upfront Technically Resectable CRLMs

**Figure 2:**
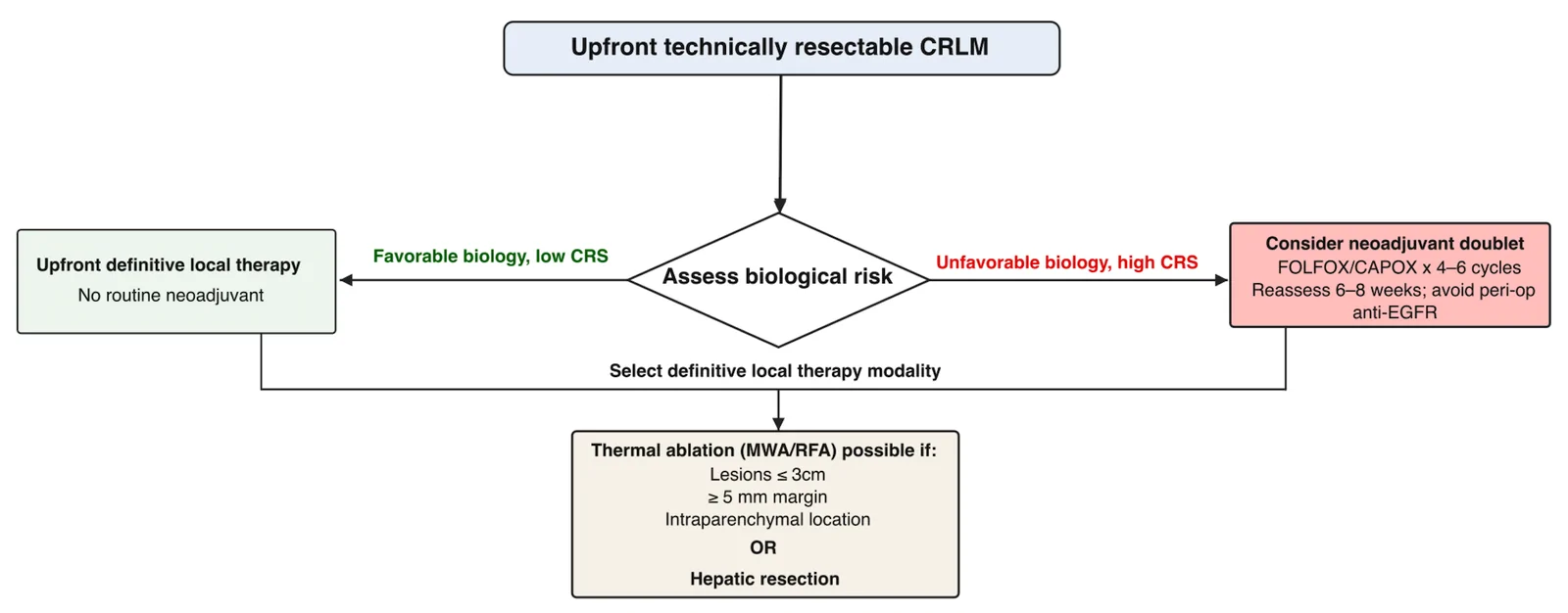
Approach to upfront technically resectable CRLMs. CRLMs, colorectal liver metastases; CRS, clinical risk score; FOLFOX, 5-fluorouracil, leucovorin, and oxaliplatin; CAPOX, capecitabine and oxaliplatin; EGFR, epidermal growth factor receptor; MWA, microwave ablation; RFA, radiofrequency ablation.

Upfront resectable disease refers to cases in which complete clearance of hepatic metastatic burden is achievable in a single stage resection and/or ablation, with preservation of the requisite technical parameters described previously. Selection of the local control modality is determined primarily by technical considerations, including complete clearance, R0 margin feasibility, and preservation of the vascular and biliary anatomy, which applies in both favorable and unfavorable biology. In the setting of upfront technically resectable CRLMs, the use of perioperative systemic therapy is principally guided by biological risk (Figure 2).

### 5.1. Approach to Perioperative Systemic Therapy

The threshold for perioperative oxaliplatin-based doublet chemotherapy (5-fluorouracil, leucovorin, and oxaliplatin [FOLFOX] or capecitabine and oxaliplatin [CAPOX]) is adjusted according to clinical risk and to minimize recurrence [10,16,17,62].

In the landmark EORTC 40983 trial, patients with resectable CRLMs were randomized to surgery alone or to surgery with perioperative FOLFOX given as six cycles before and six cycles after resection [63]. The addition of chemotherapy increased the progression-free survival at 3 years from 28.1% to 36.2% without an improvement in OS (median OS 61.3 vs. 54.3 months in the chemotherapy vs. surgery alone) [62,63]. The randomized JCOG0603 trial, which was terminated early at a planned interim analysis when the primary endpoint of DFS was met, reached a similar conclusion for adjuvant chemotherapy alone. This study demonstrated that post-hepatectomy mFOLFOX6 significantly improved DFS compared to hepatectomy alone (5-year DFS: 49.8% vs. 38.7%; HR: 0.67, *p* = 0.006), but not overall survival, which numerically favored hepatectomy alone (5-year OS 83.1% vs. 71.2%; HR 1.25, 95% CI 0.78–2.0) [64].

The routine use of neoadjuvant chemotherapy for clearly resectable CRLMs remains debated, with mixed evidence. In a multicenter propensity-matched cohort of resectable, liver-only disease, upfront surgery was associated with superior OS compared with neoadjuvant chemotherapy (median OS 82.3 vs. 57.3 months; *p* = 0.029) and no improvement in DFS [65]. These data suggest immediate local treatment adequately addresses recurrence in low-risk presentations. A prospective study by Ichida et al. demonstrated favorable 3- and 5-year OS (83% and 74%, respectively) after upfront surgery for clearly resectable CRLMs, outcomes that are comparable to series using neoadjuvant therapy in similar patients [66]. Additional retrospective analyses reached the same conclusions, finding no survival advantage for neoadjuvant chemotherapy over upfront surgery in low-risk cohorts [67,68].

### 5.2. Clinical Risk and Tumor Biology

#### 5.2.1. Favorable Biology

In the absence of adverse prognostic features identified by clinical risk scores, the disease is considered to reflect favorable biology. After confirming both technical feasibility and favorable biology, upfront surgery and/or ablation is generally preferred. Although neoadjuvant therapy may test biological responsiveness and facilitate margin-negative resection, these potential advantages pertain primarily to more advanced or borderline presentations rather than to clearly upfront resectable disease. Randomized trials have not demonstrated a survival benefit for routine neoadjuvant use in this setting [62]. Therefore, patients with upfront technically resectable disease and favorable biology should proceed with definitive local therapy first.

#### 5.2.2. Unfavorable Biology

The case can be made, however, for selective neoadjuvant courses when biology is unfavorable or tumor burden threatens R0 resection rather than considering them as routine use in all resectable cases. This is particularly relevant for patients harboring co-mutations such as RAS/TP53, where the persistent deleterious effect on recurrence and the association with micrometastases beyond visible tumor margin strengthen the rationale for preoperative systemic therapy [42]. A retrospective propensity-matched analysis by Chen et al. showed that neoadjuvant chemotherapy was associated with improved OS in patients with resectable CRLMs with high clinical risk [69]. The study demonstrated that the neoadjuvant group had significantly better 1-year, 3-year, and 5-year OS rates (96.4%, 68.5%, and 50.6%, respectively) compared to the upfront surgery group (94.4%, 50.2%, and 35.8%, respectively; *p* = 0.048) [69]. A separate propensity-matched study found that among patients with upfront resectable CRLMs and poor prognostic indicators, neoadjuvant chemotherapy may be beneficial in improving DFS (18.2 months vs. 7.5 months; *p* = 0.003) [65]. Similarly, patients with high-risk features that received neoadjuvant chemotherapy had improved median and 5-year OS compared to patients who underwent upfront surgery [67,68].

Taken together, this evidence supports the approach of upfront surgery for clearly resectable CRLMs and reserving neoadjuvant chemotherapy for patients with unfavorable prognostic features (Table 2).

### 5.3. Approach to Definitive Local Therapy

#### 5.3.1. Thermal Ablation

Local treatment refers to definitive management with either surgical resection or thermal ablation when delivered with curative intent. Within this framework, several studies have evaluated how ablation compares to resection in appropriately selected patients. In the COLLISION trial—the first phase 3 randomized controlled trial comparing these modalities—patients with up to 10 lesions measuring ≤ 3 cm were randomized to thermal ablation or resection; ablation demonstrated non-inferior OS with significantly fewer adverse events (19% vs. 46%; *p* < 0.0001) [73]. Additionally, recurrence rates at 28.9 months were comparable between the two groups (ablation—12% vs. resection—14%), and 95% of cases showed complete treatment success [73]. Similarly, the MAVERRIC prospective cohort study reported a local tumor progression rate of 17% after ablation, with a secondary efficacy rate of 92% following retreatment, highlighting the effectiveness of both approaches in managing CRLMs [74].

Optimal case selection and technique are essential to obtain these results, with the best performance seen for intraparenchymal targets where an ablation margin of at least 5 mm can be secured [73,74]. Subcapsular lesions in which a 5 mm ablation margin cannot be obtained and tumors abutting large vessels where they experience perfusion-mediated cooling are less favorable [75]. Tumors in these locations risk an ablation margin of <5 mm, which is associated with higher local recurrence and tumor progression [73,74]. While ablation can yield outcomes comparable to resection in select situations, anatomic constraints must be considered to maximize the likelihood of durable local control.

Microwave ablation (MWA) has largely supplanted radiofrequency ablation (RFA) in contemporary practice due to faster ablation times, larger and more predictable ablation zones, and reduced susceptibility to the heat-sink effect near larger vessels. Meta-analyses demonstrate similar OS, DFS, and complication rates between the two modalities, though MWA achieves lower rates of local tumor progression [76,77]. Society guidelines suggest that either MWA or RFA can be safe and feasible in appropriately selected patients with CRLMs < 5 cm, though evidence quality remains low [78].

#### 5.3.2. Hepatic Resection

Accordingly, when lesions are not amenable for ablation, surgical resection remains the treatment option of choice. Hepatectomy approaches encompass both parenchymal sparing and formal anatomic resections.

Intraoperative ultrasound (IOUS) remains a routine adjunct during hepatic resection for CRLM. It is frequently used to guide the parenchymal transection plane and confirm the adequacy of the intended margin. IOUS allows reassessment of lesion number and relationship to intrahepatic vascular structures and frequently detects additional lesions not seen on preoperative cross-sectional imaging, identifying occult metastases in approximately 10% of patients [79].

An anatomic resection, as initially outlined according to the Brisbane terminology, refers to the complete removal of liver parenchyma associated with the respective portal territory guided by anatomical landmarks [80,81]. These resections include segmentectomy, sectionectomy, hemihepatectomy, and trisectionectomy, which are each defined based on portal vein inflow [80,81]. Since this terminology was introduced, parenchymal sparing resections (PSRs) have been increasingly implemented as the boundaries of technical resectability have expanded [82]. As the name suggests, PSRs are liver resections with preservation of as much liver parenchyma as possible.

PSR aims to preserve as much functional liver as possible, and its growing significance is reflected in the updated Tokyo terminology, which now formally defines subsegmentectomy as removal of a discrete subportion of a Couinaud segment based on subsegmental portal inflow [83]. Under this nomenclature, parenchymal-preserving resections that follow portal territories are therefore considered anatomic resections and reinforces PSR as a principle of safe liver surgery [83].

Since this technique has become more widely adopted, several studies have demonstrated equivalent oncologic outcomes, decreased postoperative morbidity, and similar recurrence rates compared to anatomic or major hepatectomy [84,85,86,87]. Importantly, the margin widths and rates of R0 resection were similar between PSR and non-PSR, confirming the oncological adequacy of PSR [86,87]. However, PSR facilitates repeat resection in the setting of recurrence improving salvageability and provides an overall survival advantage [86,87,88].

Whether the extent of anatomic resection influences outcomes in the setting of unfavorable tumor biology is a critical consideration. Retrospective analyses stratified by KRAS status report that in KRAS-mutated disease, anatomic resection is associated with longer median DFS (33.8 vs. 10.5 months, *p* < 0.001) and lower intrahepatic recurrence (28% vs. 53%, *p* = 0.012) compared with non-anatomic resection [89]. In contrast, a separate propensity-matched cohort with known RAS status found no differences in OS or DFS between anatomic and nonanatomic approaches [90].

While these findings derive from retrospective cohorts that are vulnerable to selection bias, randomized comparisons are not available. There is therefore no definitive evidence necessitating anatomic resection in patients with specific genotypes, but adjusting margins may be appropriate for those with genomic profiles associated with higher risk of satellite lesions and narrow margins.

#### 5.3.3. Optimal Margins

The 1 cm margin recommended by Ekberg et al. in 1986 has been steadily reevaluated, with contemporary chemotherapy and improvement in surgical technique allowing narrower margins in selected patients [91,92].

More recent evidence, drawn predominantly from large retrospective cohorts, favors a biology-informed rather than a width-driven margin strategy. In a large retrospective series evaluating patients that underwent R0-intent (≥1 mm) resection, local recurrence occurred in 7% of patients and did not vary by margin width [93]. RAS/TP53 co-mutation was associated with both increased intrahepatic and overall recurrence but did not determine margin-site failure [93]. Similar findings were reported from two high-volume centers, indicating that R1 resection did not lead to increased local recurrence rates compared to R0 resection and did not negatively impact OS when compared to other recurrence patterns [94]. However, R1 status was associated with a generally worse OS, suggesting it may act as a marker of more advanced disease rather than a direct cause of poorer outcomes [94]. While R1 resections are associated with worse OS and DFS, this was not observed in patients with favorable tumor biology, such as those with a good response to chemotherapy or RAS wild type (RAS-wt), where outcomes were comparable to R0 resections (5-year OS: 66.4% for R0 vs. 65.2% for R1, *p* = 0.884) [95]. A retrospective study evaluating the impact of surgical margin width on long-term outcomes in CRLMs emphasized the critical role of tumor biology, specifically KRAS mutational status, in evaluating the impact of surgical margins on prognosis [96]. Among patients with KRAS-wt tumors, achieving a margin width of 1–4 mm was associated with improved OS compared to R1 resections (HR = 0.45, *p* = 0.014), but wider margins (5–9 mm or ≥10 mm) did not confer additional survival benefits (*p* > 0.05) [96]. In contrast, in patients with KRAS-mutated (KRAS-mut) tumors, margin width—including achieving a 1 cm margin—did not improve OS, as outcomes were comparable between R0 and R1 resections (5-year OS: 47.9% for R0 vs. 40.7% for R1, *p* = 0.470) [96].

These findings highlight the importance of aligning surgical margin strategies with the underlying tumor biology rather than applying a fixed universal margin width. The operative aim is a negative margin whenever possible while accepting narrow margins (1–4 mm) if necessary, in a setting of favorable biology and without expectation of additional benefit from wider excisions in genomically adverse disease.

## 6. Management of Synchronous Disease

Synchronous CRLMs are hepatic metastases identified at or before the diagnosis of the primary colorectal tumor, although some definitions include diagnosis of metastases up to 12 months [18]. Compared with metachronous presentation, synchronous disease often reflects more aggressive biology and portends worse prognosis [18]. Management hinges on a coordinated multidisciplinary assessment with high-quality staging imaging and determination of both technical and oncologic resectability. Management strategies for synchronous CRLMs differ based on patient symptoms and the extent of disease burden, with surgical timing and sequence summarized according to the following scenarios: resectable CRLMs with asymptomatic primary, borderline or initially unresectable CRLMs with asymptomatic primary, resectable CRLMs with symptomatic primary, and borderline or initially unresectable CRLMs with symptomatic primary.

In cases where the primary tumor presents with symptoms such as bleeding, obstruction, or perforation, initial management should focus on addressing the primary lesion urgently before proceeding to treatment of metastatic disease. For initially unresectable liver disease, the treatment is directed in conversion with systemic therapy, followed by hepatic resection and subsequent management of the primary tumor [18]. In contrast, the management of resectable disease is characterized by substantial variability, including whether preoperative chemotherapy is administered, how treatment is sequenced, and whether colorectal and hepatic resections are undertaken simultaneously or staged.

### 6.1. Surgical Sequencing in Resectable Synchronous Disease—Simultaneous vs. Staged Resection

Across comparative studies, no single operative sequence is uniformly superior for all patients with resectable synchronous CRLM. A systematic review and meta-analysis including 2884 patients reported no difference in 5-year OS between simultaneous and staged resections [97]. Simultaneous resections were associated with higher 3-year DFS (OR 1.67, 95% CI 1.28–2.17; *p* = 0.0001) but also higher rates of major complications [97]. In contrast, a large international registry analysis of 7360 patients suggests sequence strategy may carry importance in select patients [98]. Among patients with multiple bilobar metastases, a liver-first sequence was independently associated with superior survival (3-year OS 65.9% vs. 60.4% for colorectal-first vs. 54.4% for simultaneous), whereas outcomes were similar across approaches for solitary or unilobar disease [98].

Depending on the extent of the operation, perioperative risk increases when major resections are combined. A single-institution series of 273 synchronous resections demonstrated total morbidity, major morbidity, and 90-day mortality differed across operative pairing [99]. Combined major liver/major colorectal resections had the highest rates (58.3%, 54.2%, and 16.7%, respectively), followed by major liver/minor colorectal (44.2%, 23.3%, 4.7%), minor liver/major colorectal (40.2%, 14.1%, 1.1%), and minor liver/minor colorectal (32.5%, 14.0%, 3.5%) [99]. Complications delayed adjuvant chemotherapy in 17.6% of patients and precluded it in 9.3% of patients, which correlated with a nearly six-fold higher risk of death (HR 5.91) [99]. A separate retrospective cohort study of over 1000 patients similarly found that simultaneous resection was associated with more major complications (OR 1.89, *p* = 0.001), a shorter median OS (40 vs. 78 months, *p* < 0.001), and reduced receipt of chemotherapy (76% vs. 91%, *p* < 0.001), compared with staged resections [100].

Randomized evidence is limited to the METASYNC trial, a small, open-label study (n = 105) that compared simultaneous vs. staged resection for resectable synchronous CRLMs [101]. Major postoperative complications did not differ between the two groups (48.7% simultaneous resection vs. 45.7% staged resection) [101]. Early survival favored simultaneous surgery (2-year OS 87.2% vs. 69.6%; 2-year DFS 35.9% vs. 17.4%; both *p* = 0.05), yet with longer follow-up there was no statistically significant difference in median OS or DFS (5.9 vs. 3.9 years, *p* = 0.07; 1.3 vs. 1.0 years, *p* = 0.57) [101].

**Figure 3:**
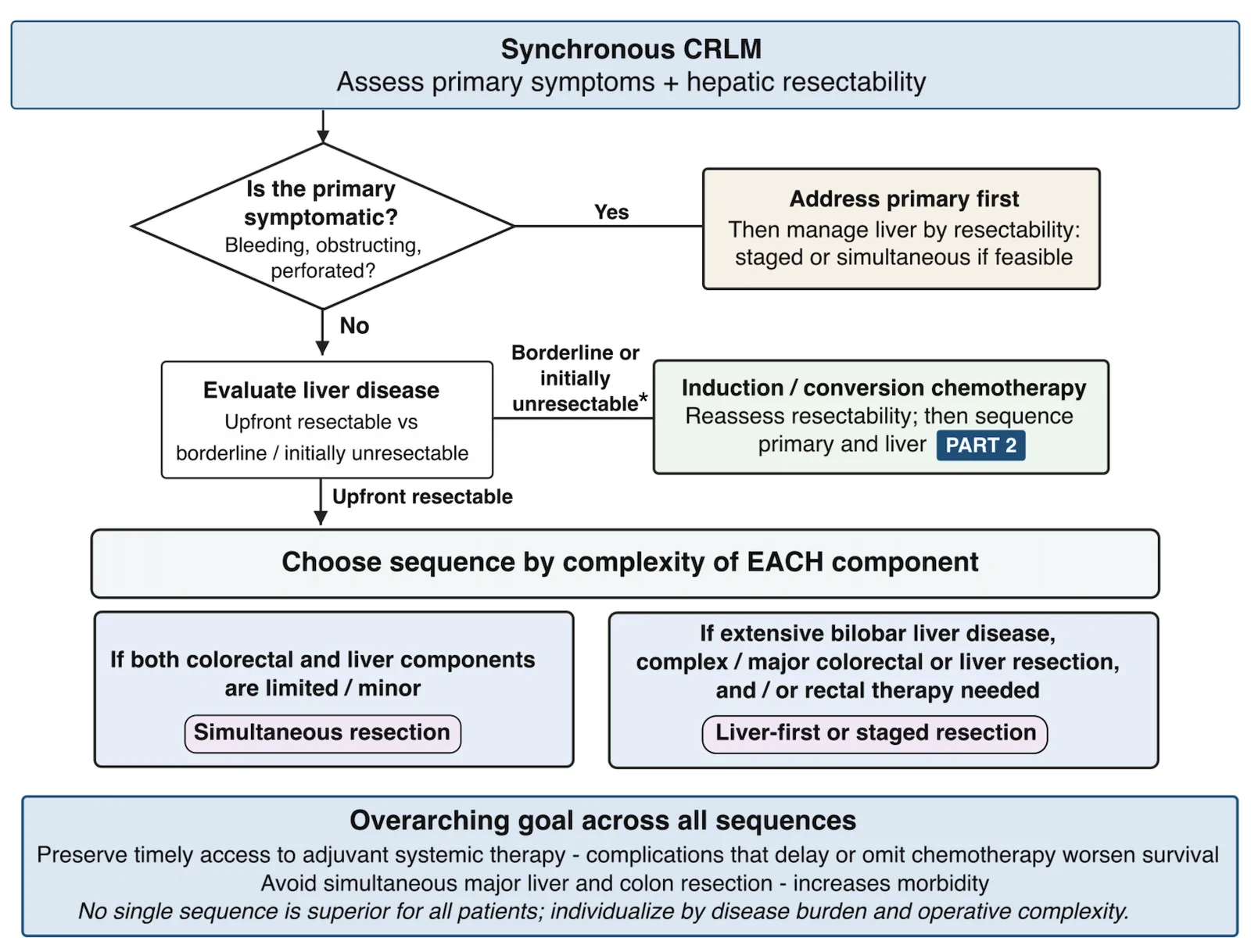
Approach to management of synchronous CRLMs. CRLMs, colorectal liver metastases. * Detailed management of both borderline resectable and initially unresectable CRLMs are discussed in Part 2 of this review.

Taken together, these data support simultaneous resection in carefully selected patients when the hepatic component is limited and the colorectal operation is not complex, favor a liver-first strategy for patients with extensive bilobar disease or when rectal therapy must be integrated, and recommend staging when either component would be major to minimize morbidity and preserve the timely delivery of systemic therapy (Figure 3) [18,98].

### 6.2. Treatment Sequencing in Resectable Synchronous Disease—Neoadjuvant Chemotherapy vs. Upfront Resection

Contemporary retrospective data shows that neoadjuvant chemotherapy does not confer a consistent survival advantage in resectable synchronous CRLMs and may increase perioperative morbidity. In a single-center retrospective cohort study comparing combined colorectal and liver resections with and without neoadjuvant chemotherapy, preoperative treatment was associated with greater intraoperative blood loss (median 550 vs. 200 mL), higher morbidity (32.9% vs. 24.7%; *p* = 0.043), and longer hospitalization (12 vs. 9 days) [102]. The median OS, DFS, and recurrence rates were similar in both groups [102]. On multivariable analysis, adverse biology (i.e., right-sided primaries, RAS mutation, and clinical risk scores ≥ 3) drove prognosis. Subgroup analysis, however, demonstrated that patients with high clinical risk who received neoadjuvant chemotherapy exhibited improved DFS compared to those with high clinical risk who underwent upfront surgery (*p* = 0.04) [102].

Findings from the LiverMetSurvey registry reinforce this pattern. In an analysis of 1301 patients with resectable synchronous CRLMs, overall survival did not differ between upfront surgery and ≥3 cycles of neoadjuvant chemotherapy (3-year OS 74% vs. 72%; *p* = 0.72), whereas DFS favored upfront surgery (3-year DFS 33% vs. 23%; *p* < 0.001) [103]. Neoadjuvant treatment was not an independent determinant of OS or DFS; rather, higher nodal stage, elevated CEA, and omission of postoperative chemotherapy predicted inferior outcomes [103].

Two liver-specific considerations further argue against prolonged preoperative chemotherapy when the disease is already resectable and are discussed in detail in Part 2 of this review. Chemotherapy-associated liver injury is regimen-specific. Oxaliplatin is associated with sinusoidal obstruction syndrome and irinotecan with steatohepatitis, increasing with the number of cycles and associated with postoperative morbidity and mortality [104,105]. Additionally, effective chemotherapy can render metastases radiographically undetectable, commonly known as “disappearing” liver metastases, and a substantial proportion of these sites harbor viable tumors or recur if not resected, complicating operative planning when the surgical target is lost [106].

### 6.3. Minimally Invasive Approaches

The operative approach increasingly informs the above sequencing decision. The 2017 Southampton consensus guidelines endorsed laparoscopic liver resection as standard practice for lesions in favorable segments and as a valid option in expert centers for more complex resections, including simultaneous colorectal and peripheral hepatic resection [107]. Across randomized and matched cohorts, minimally invasive hepatectomy yields less blood loss, few complications and shorter length of stay with equivalent R0, DFS, and OS compared with open surgery, for both staged and simultaneous resections [108,109]. Importantly, the minimally invasive approach shortens the interval to adjuvant systemic therapy, an outcome most consistently linked to survival [108].

Collectively, these data support a selective and individualized strategy for resectable synchronous CRLMs. In favorable-risk presentations with limited hepatic burden, upfront resection followed by timely postoperative chemotherapy minimizes perioperative morbidity without sacrificing long-term outcomes. Preoperative chemotherapy should be reserved for patients with adverse clinical features or threatened resection margins, where a short course may improve operability or refine biologic risk. Management should avoid combining major colorectal and major hepatic procedures, as this may compromise recovery and delay adjuvant therapy. Given the retrospective design and potential selection biases of the available studies, and the limitations of the only randomized trial, prospective trials are warranted to more precisely identify subgroups that derive benefit from neoadjuvant therapy in this setting.

## 7. Discussion and Future Directions

The approach to treatment of CRLMs has evolved over the last few decades and has moved beyond a model in which surgery was reserved for a small subset of patients with limited disease. As a result, many patients who would previously have been considered unresectable are now candidates for potentially curative surgery. This review evaluates the evidence that supports the shift from anatomy-driven to biology-informed decision-making in CRLM, though several conclusions rest on retrospective and propensity-matched data with inherent limitations in confounding control and generalizability.

### 7.1. Surgical Strategy: Biology over Anatomy

Historically, resectability was defined almost exclusively by technical criteria—lesion number, size, distribution, and the feasibility of achieving negative margins with adequate FLR. While these parameters remain essential, contemporary evidence increasingly demonstrates that tumor biology exerts an equal or greater influence on long-term outcomes, as detailed in the margin data above (Section 5.3.3). The prognostic significance of R0 versus R1 resection depends less on margin width than on biological context and supports an operative aim of a negative margin when technically achievable, without expecting wider margins to compensate for adverse biology [94,95,96]. However, this biology-informed margin strategy rests entirely on retrospective evidence. Prospective studies correlating margin width, molecular profile, and site-specific recurrence patterns are needed. Similarly, whether anatomic resection confers a meaningful advantage over non-anatomic approaches in KRAS-mutated disease remains unresolved, with conflicting data and no randomized comparisons available. The growing adoption of PSR reflects a strategic prioritization of salvageability—preserving a functional liver to enable re-resection at recurrence—over anatomic resection at the index operation. Given that recurrence affects approximately 70% of patients, the ability to offer repeat local therapy may be as important to long-term survival as the completeness of the initial resection.

### 7.2. Perioperative Chemotherapy: An Unresolved Question

The role of perioperative systemic therapy in upfront resectable disease remains one of the most debated questions in CRLM management. Despite decades of investigation, no randomized trial has demonstrated an OS benefit for perioperative chemotherapy in resectable CRLMs. The evidence supports a risk-stratified approach—upfront surgery for favorable biology, selective neoadjuvant courses for high-risk presentations—rather than a uniform perioperative strategy [65,66,67,68,69]. The absence of a dedicated randomized trial comparing these approaches in high-risk populations, stratified by clinical risk score and molecular profile, remains the most critical evidence gap. Emerging biomarker data further challenge the concept of a “one-size-fits-all” approach to systemic therapy. Postoperative circulating tumor DNA (ctDNA) detection is strongly predictive of recurrence after hepatectomy, and the CIRCULATE-Japan GALAXY study demonstrated that adjuvant chemotherapy improved OS in patients with molecular residual disease but not in those who were ctDNA-negative, suggesting that the benefit of systemic therapy may depend on postoperative disease biology rather than clinicopathologic risk factors alone [110,111,112]. Whether ctDNA can similarly identify patients most likely to benefit from preoperative chemotherapy or support omission of systemic therapy remains unknown and requires prospective validation in CRLM.

### 7.3. Synchronous Disease: Variability Without Consensus

As outlined in Section 6, no sequencing strategy has consistently demonstrated superior long-term survival in synchronous disease, and outcomes appear driven less by operative sequence than by the ability to deliver planned adjuvant chemotherapy [99,100,103]. Adequately powered randomized trials comparing these approaches are warranted, particularly as total neoadjuvant therapy (TNT) for rectal cancer introduces sequencing considerations not yet prospectively studied. Preliminary data suggest that patients developing liver metastases during or after TNT may have worse OS after hepatectomy and higher rates of chemotherapy-associated liver injury, raising questions that require further investigation [113].

## 8. Conclusions

The key principles that emerge from this review are: (1) resectability should be assessed through the integration of patient fitness, technical feasibility, and tumor biology rather than any single criterion; (2) perioperative systemic therapy should be risk-stratified, with upfront surgery preferred for favorable biology and selective neoadjuvant courses reserved for high-risk presentations; (3) surgical margin strategy should be biology informed rather than width-driven; (4) the approach to synchronous disease should be individualized based on the complexity of both the colorectal and hepatic components, with the goal of preserving timely access to systemic therapy; and (5) multidisciplinary evaluation is essential at every decision point.

In part 2 of this review, the focus shifts to the management of borderline resectable, initially unresectable, and never resectable CRLMs—clinical scenarios that require escalating complexity in both surgical technique and systemic therapy [20].

## Figures and Tables

**Table 1:** Different clinical risk scores.

	CRS Components (Each Risk Factor Is 1 Point)	Risk Group
Nordlinger [36]	Age > 60 Serosal invasion of primary tumor (≥pT3) Lymph node positive primary tumor Disease-free interval < 24 months Number of liver metastases > 3 Largest liver metastasis > 5 cm	Low: 0–2 points Intermediate: 3–4 points High: 5–6 points
Fong [35]	Lymph node positive primary tumor Disease-free interval < 12 months Number of liver metastases > 1 Largest liver metastasis > 5 cm CEA >200 ng/mL	Low: 0–2 points High: 3–5 points
Nagashima [37]	Serosal invasion of primary tumor (≥pT3) Lymph node positive primary tumor Number of liver metastases ≥ 2 Largest liver metastasis ≥ 5 cm Resectable extrahepatic metastases	Low: 0–1 points Intermediate: 2–3 points High: 4–5 points
Konopke [38]	Synchronous CRLMs Number of liver metastases ≥ 4 CEA ≥ 200 ng/mL	Low: 0 points Intermediate: 1 point High: 2–3 points

CRS, clinical risk score; CEA, carcinoembryonic antigen; CRLMs, colorectal liver metastases.

**Table 2:** Approach to systemic therapy based on resectability *.

Setting	Molecular/ Clinical Subset	Preferred Regimen	Key Evidence	Practical Notes
**Upfront** **resectable**	Favorable biology, low CRS	Upfront surgery ± adjuvant doublet (FOLFOX/CAPOX)	No OS gain with perioperative or adjuvant chemotherapy; comparable outcomes with surgery alone (EORTC 40983, JCOG0603)	Proceed to definitive local therapy; adjuvant doublet optional
	Unfavorable biology, high CRS	Consider perioperative doublet (FOLFOX/CAPOX)	Retrospective and PSM data suggest OS or DFS benefit in high-risk subgroups No dedicated RCT Avoid perioperative anti-EGFR (New EPOC) [70]	Limit to 4–6 cycles, reassess at 6–8 weeks
	MSI-H/dMMR ^‡^	Pembrolizumab (PD-1 monotherapy) or nivolumab + ipilimumab	PFS advantage vs. chemo (KEYNOTE-177, CheckMate-8HW) [71,72]	If chosen pre-op, keep course short with defined surgical timing

* Regimen for borderline resectable and initially unresectable disease is detailed in Part 2 [20]. ^‡^ MSI-H/dMMR status is determined by mismatch-repair immunohistochemistry, MSI-PCR, or NGS. Immunotherapy eligibility in CRC is based on MSI-H/dMMR status alone. CRS, clinical risk score; OS, overall survival; PSM, propensity-score matched; DFS, disease-free survival; RCT, randomized controlled trial; MSI-H, microsatellite instability-high; dMMR, mismatch repair deficient; PD-1, programmed cell death protein; PFS, progression free survival; PCR, polymerase chain reaction; NGS, next-generation sequencing; CRC, colorectal cancer.

## Data Availability

No new data were created or analyzed in this study. Data sharing is not applicable to this article.

## Supplementary Materials

The following supporting information can be downloaded at: https://www.mdpi.com/article/10.3390/cancers18193165/s1, Table S1: Scale for the Assessment of Narrative Review Articles—SANRA.

## Abbreviations

The following abbreviations are used in this manuscript:

anti-EGFR	Anti–epidermal growth factor receptor
ASCO	American Society of Clinical Oncology
CAPOX	Capecitabine and oxaliplatin
CEA	Carcinoembryonic antigen
CRC	Colorectal cancer
CRLM	Colorectal liver metastasis
CRS	Clinical risk score
CT	Computed tomography
DFS	Disease-free survival
dMMR	Deficient mismatch repair
DWI	Diffusion-weighted imaging
EORTC	European Organisation for Research and Treatment of Cancer
FDG	Fluorodeoxyglucose
FLR	Future liver remnant
FOLFOX	Folinic acid, 5-fluorouracil, and oxaliplatin
Gd-EOB-DTPA	Gadolinium ethoxybenzyl diethylenetriamine pentaacetic acid
HAI	Hepatic artery infusion
HIPEC	Hyperthermic intraperitoneal chemotherapy
HR	Hazard ratio
IOUS	Intraoperative ultrasound
MDT	Multidisciplinary team
MeSH	Medical Subject Headings
MRI	Magnetic resonance imaging
MSI-H	Microsatellite instability-high
MWA	Microwave ablation
OS	Overall survival
PD-1	Programmed cell death protein 1
PET	Positron emission tomography
PSM	Propensity-score matched
PSR	Parenchymal-sparing resection
RCT	Randomized controlled trial
RFA	Radiofrequency ablation
SANRA	Scale for the Assessment of Narrative Review Articles
TNT	Total neoadjuvant therapy
